# Oral Supplementation with Bovine Colostrum Decreases Intestinal Permeability and Stool Concentrations of Zonulin in Athletes

**DOI:** 10.3390/nu9040370

**Published:** 2017-04-08

**Authors:** Maciej Hałasa, Dominika Maciejewska, Magdalena Baśkiewicz-Hałasa, Bogusław Machaliński, Krzysztof Safranow, Ewa Stachowska

**Affiliations:** 1 Department of Biochemistry and Human Nutrition, Pomeranian Medical University, Szczecin 70-204, Poland; domi.maciejka@wp.pl (D.M.); ewast@pum.edu.pl (E.S.); 2 Department of General Pathology, Pomeranian Medical University, Szczecin 70-111, Poland; poziomka@pum.edu.pl (M.B.-H.); machalin@pum.edu.pl (B.M.); 3 Department of Biochemistry and Medical Chemistry, Pomeranian Medical University, Szczecin 70-111, Poland; chrissaf@mp.pl

**Keywords:** intestinal permeability, stool zonulin concentration, colostrum supplementation

## Abstract

Increased intestinal permeability has been implicated in various pathologies, has various causes, and can develop during vigorous athletic training. Colostrum bovinum is a natural supplement with a wide range of supposed positive health effects, including reduction of intestine permeability. We assessed influence of colostrum supplementation on intestinal permeability related parameters in a group of 16 athletes during peak training for competition. This double-blind placebo-controlled study compared supplementation for 20 days with 500 mg of colostrum bovinum or placebo (whey). Gut permeability status was assayed by differential absorption of lactulose and mannitol (L/M test) and stool zonulin concentration. Baseline L/M tests found that six of the participants (75%) in the colostrum group had increased intestinal permeability. After supplementation, the test values were within the normal range and were significantly lower than at baseline. The colostrum group Δ values produced by comparing the post-intervention and baseline results were also significantly lower than the placebo group Δ values. The differences in stool zonulin concentration were smaller than those in the L/M test, but were significant when the Δ values due to intervention were compared between the colostrum group and the placebo group. Colostrum bovinum supplementation was safe and effective in decreasing of intestinal permeability in this series of athletes at increased risk of its elevation.

## 1. Introduction

Recognition of the influence of gut status on a variety of body functions has spurred investigation of the role of intestinal homeostasis [1]. The effect of the gut physiology on the immune system is recognized [2]. This may help explain intestinal involvement in generation of allergies and, more recently, auto-aggressive immune responses [3].

Several mechanisms have been proposed to explain the influence of intestinal malfunction on the immune system dysregulation [4,5,6], the most compelling of which point to involvement of intestinal permeability status [7,8,9,10]. The relationship of zonulin, which is a physiological permeability regulator, and the development of celiac disease described by Fasano et al. was the first established pathogenetic axis for an autoimmune disease [11]. This discovery encouraged additional research, demonstrating that reported increased gut permeability may favor development of type 1 diabetes mellitus, Hashimoto’s thyroiditis, autoimmune hepatitis, and connective tissue autoimmune diseases [12]. Other pathologies, including a variety of hypersensitivity disorders, may develop in association with increased gut permeability, but the current supporting evidence is weak. Nevertheless, the currently available evidence indicates that increased permeability of the intestinal barrier is potentially harmful to health.

Two approaches to maintaining intestinal barrier permeability within physiological limits are available. One very effective, though impractical, approach relies on avoiding factors like gluten, nonsteroidal anti-inflammatory drugs, and intense physical activity that may damage the structure of the barrier or excessively upregulate permeability [11,13,14]. Another approach is to restore and maintain physiological permeability by dietary or pharmacological intervention. Blocking the zonulin receptor has been investigated by Fasano [15], and that method seems promising in cases of increased permeability developing because of pathologically upregulated zonulin [16]. Other approaches utilizing glutamine and probiotic supplementation have also been studied [17,18,19]. It is very likely, however, that zonulin-independent causes of permeability increase are also involved in human pathology, such as inflammatory damage to the gut epithelial structure [13]. This will require other approaches to restore the integrity of the intestinal barrier.

Colostrum bovinum has been reported to positively influence gut status in both human and animal studies [20]. Well-established causes of these effects include participation in restoring the composition of the intestinal microbiome and support of the healing of damaged mucosa [21,22,23]. It has also been suggested that colostrum may support the reduction of hypersensitivity associated with allergies and autoimmunity [24]. These latter claims, although mostly unsubstantiated, generally point to health improvements related to gut changes associated with colostrum supplementation.

We hypothesized that hypersensitivity-related health problems can be alleviated by colostrum supplementation and that this effect might be attributed to normalization (decrease) of intestinal permeability. To test this, we investigated the changes in gut permeability in response to colostrum bovinum in a group of actively training athletes. We chose athletes because they are known to have increased intestinal permeability [25,26], which is also in line with our previous unpublished results. We tested gut permeability before and after a relatively brief 20-day supplementation with a typical twice daily dose of 500 mg colostrum bovinum.

## 2. Materials and Methods

### 2.1. Recruitment

A group of 16 healthy male volunteers between 20 and 43 years of age (median = 26 years, mean = 27.5 years) was recruited randomly from 40 professional mixed martial arts fighters in active training during the middle of the competitive season. Written informed consent was obtained from each participant. The participants were free of any symptomatic acute disease and were physically fit to participate in extensive athletic training. On inclusion, all participants received a physical examination that included measurement of lean body mass (LBM), total body water (TBW), and peripheral body fat (PBF). The characteristics are shown in Table 1.

### 2.2. Double-Blind Study

#### 2.2.1. Blinding and Initial Assessment of Participants

On trial inclusion (day 0), the 16 participants were randomly allocated by a table of random numbers to two equal groups to receive placebo or colostrum. The administrator did not participate in the participants’ assessment or laboratory testing, and the investigators were blinded to the group allocation. Participants were tested (stool and urine collection) on day 0 for baseline intestinal permeability by a differential sugar absorption test and assay of stool zonulin concentration. A self-reporting questionnaire was administered on day 0 to inquire about participant health, lifestyle, food habits, and athletic activity. The participants also responded to questions about digestive tract discomfort or symptoms, the character and the frequency of stools and recent use of medications, including nonsteroidal anti-inflammatory drugs and antibiotics.

#### 2.2.2. Supplementation: Days 1–20

Freeze-dried whole bovine colostrum obtained within 2 h of calf delivery (Genactiv Sp. z o.o., Poznań, Poland) was packaged in unlabeled pouches (500 mg colostrum bovinum and 500 mg desiccated banana). The dose of 500 mg of colostrum powder is within the range of a typical single supplementation dose suggested by various suppliers. Identical pouches (500 mg of dehydrated whey and 500 mg of desiccated banana) were used as the placebo. The difference between colostrum and whey used in the trial was in expected biological activity of bioactive components contained in them. While roughly 250 various bioactive components (mostly proteins) present in colostrum were preserved and undamaged by heat in the freeze-drying process where temperatures reach no more than 40 °C, the whey, which is several folds less biologically active, was spray-dried, with temperatures reaching 160 °C. The latter process is expected to damage function of most of the originally bioactive proteins.

The trial administrator distributed packages of 40 colostrum or placebo pouches to participants in the intervention and control groups. The participants were asked to start using the provided test substances on day 1 of the trial. The schedule was one pouch orally in the morning and one in the evening; both doses were taken 30 min before a meal. Supplementation was scheduled to last for 20 days. During the supplementation period, the participants were asked to record self-observations and take note of unusual or adverse reactions, if any occurred. The original questionnaire was provided for that purpose, and was intended to attract participant attention to digestive tract symptoms and their general health status.

#### 2.2.3. Final Assessment: Day 22

On day 22, the participants were tested for differential sugar absorption, and assayed with respect to urine samples and stool zonulin concentration (ng/mL). The questionnaires, including the participant reports of events from the previous 22 days, were included in the final assessment.

### 2.3. Open-Label Study

Because the availability of participants was limited by their training and competition schedules, an adequate washout period could not be achieved for a blinded crossover study. Instead, we decided to perform an open-label phase of the trial only for the participants originally receiving the placebo. One participant dropped out from the original placebo group before the crossover phase because of a conflict with the competition schedule. The remaining seven placebo participants were given colostrum supplementation for 20 days starting on day 23 and tested for intestinal permeability and stool zonulin concentration on day 44. The results were compared with those previously obtained from the same patients during their participation in the placebo group of the double blind phase.

### 2.4. Differential Sugar (Lactulose/Mannitol) Absorption Test

#### 2.4.1. The Test Principle

The test depends on the difference in pathways of gut absorption utilized for lactulose and mannitol [27]. If intercellular tight junctions are damaged or relaxed, urinary excretion of lactulose, which is absorbed primarily through a paracellular pathway, increases relative to mannitol, which is transcellularly absorbed. The differential sugar absorption test can be regarded as a direct marker of intestinal permeability.

#### 2.4.2. Sugar Ingestion and Urine Collection

The test began in the morning following an 8-h fast. Each participant emptied their bladder, collected 100 mL of urine as a blank (negative control) sample, and subsequently drank 500 mL of water solution containing 7.5 g lactulose and 2 g mannitol. For the next 6 hours, participants were allowed to eat, except for specified foods (milk and dairy products, simple sugars, large doses of vitamin C and mannitol), and were asked to collect all urine passed into one container. Finally, 400-μL aliquots of urine from the collection container and from the blank sample were assayed.

#### 2.4.3. Sugar Derivatization

The 400-μL urine aliquots were mixed with 40 μL of an internal standard (myo-inositol, 20 mg/mL) and evaporated to dryness by lyophilization. Then 200 μL of anhydrous pyridine in hydroxylamine (25 mg/mL) was added, mixed, and heated to 70 °C for 1 h. The sample was centrifuged at 800 × g for 5 min and 200 μL of supernatant was collected. Sugars were silylated with 100 µL of *N*-trimethylsilylimidazole for 30 min at 70 °C and assayed by gas chromatography.

#### 2.4.4. Gas Chromatography

Gas chromatography was performed with an Agilent Technologies 7890A GC System and capillary column (15 m × 0.530 mm, 1.50 μm), (Supelco, Bellefonte, PA, USA). Chromatographic conditions included an initial temperature of 220 °C for 5 min, increased at a rate of 10 °C/min for 2 min, 5 °C/min for 4 min, and 3.5 °C/min for 4 min to a final temperature of 274 °C, which was maintained for 7 min. The total time was approximately 22 min, and hydrogen was the carrier gas. Lactulose, mannitol, and myo-inositol were identified by comparing their retention times with those of commercially available standards.

### 2.5. Stool Zonulin Assay

The concentration of zonulin in stool was determined with a competitive enzyme-linked immunosorbent assay (ELISA) kit (Immundiagnostik AG, Bensheim, Germany) following the manufacturer’s protocol. Stool samples were collected 1 day before the mannitol/lactulose test. Assay plate wells were coated with polyclonal anti-zonulin antibody; zonulin in the samples was conjugated to biotinylated zonulin tracer and then immobilized on the plate. Absorbance was measured by a photometer at 450 nm [18].

### 2.6. Reference Limits

We established internal upper reference limits of the lactulose to mannitol ratio (L/M) < 0.035 and zonulin < 30 ng/mL referring to previous studies [18], our own experience, and the statistical analysis.

### 2.7. Statistical Analysis

We performed the statistical analysis using Microsoft Excel 2010 and STATISTICA 12. The Shapiro–Wilk test was used to determine the normality of the distributions of experimental variables, and the significance of differences at different times was determined by Student’s paired sample t-test. The unpaired t-test was used to compare values of independent variables. For variables with distributions not normally distributed, the nonparametric Wilcoxon signed-rank test and Mann–Whitney U test were used for paired and unpaired comparisons, respectively. Between-group differences in frequency of treatment-associated side effects and abnormal test values were analyzed with Fisher’s exact test. Differences with *p* < 0.05 were regarded as statistically significant and are shown on the figures. Statistical power analysis was performed basing on standard deviations of baseline laboratory test results. The power of our study with eight subjects in each group was sufficient to detect with 80% probability true differences between placebo and colostrum groups equal to 0.029 for the L/M ratio and 17 ng/mL for stool zonulin concentration.

### 2.8. Bioethical Approval

The trial was performed in accordance with the protocol conditions approved by the Pomeranian Medical University Bioethics Committee (KB-0012/05/16).

## 3. Results

The interview and questionnaire data revealed that none of the participants in the colostrum group reported any important treatment-associated digestive system side effects. Four participants in the placebo group (50%) reported adverse digestive symptoms (loose stools, bloating, nausea and/or lack of appetite) but the difference in relation to the colostrum group did not reach statistical significance (*p* = 0.08). Both the colostrum and placebo group participants reported their general shape to be good/very good and remaining either the same as it was before the trial or improved after supplementation. Two participants receiving colostrum (25%) and five in the placebo group (62.5%) experienced upper respiratory tract infections during the supplementation period of the double-blind phase of the trial. The difference between groups was non-significant (*p* = 0.31).

There were no significant correlations of laboratory test results and participant history data (including dietary and health-related variables) collected by the direct interviews or questionnaires. This was probably because of the small number of participants. However, the small study sample did not prevent obtaining statistically significant between-group differences in sugar absorption and zonulin concentration.

The percentage of individuals showing higher-than-normal baseline L/M ratio (L/M ≥ 0.035) was not significantly different between the placebo and colostrum groups (50% vs. 75%, respectively, *p* = 0.61). The proportion of subjects with baseline zonulin level above the reference limit of 30 ng/mL was similarly high in placebo and colostrum groups (87.5% vs. 100%, respectively, *p* = 1).

All participants, except for two in each of the two groups, reported full compliance with the administration schedule. Those who failed to fully comply have claimed to have missed between 2 and 4 doses out of 40 supplied.

**Figure 1:**
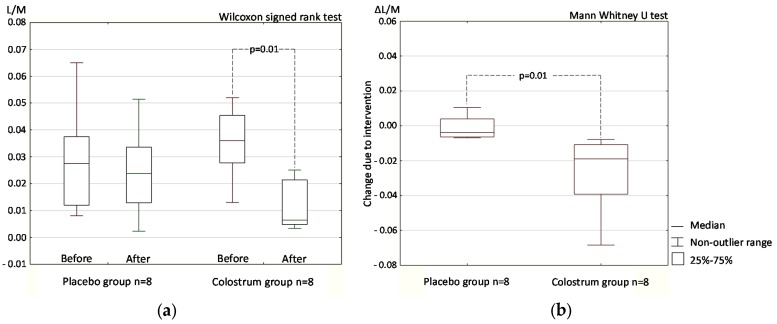
Intestinal permeability assayed by differential sugar absorption and expressed as the lactulose to mannitol ratio (L/M) in urine following placebo or colostrum supplementation (**a**); The change in permeability after vs. before intervention (Δ) within the placebo and the colostrum groups (**b**). Data from the double-blind placebo controlled phase of the trial.

Except for one participant in the colostrum supplementation with increased stool zonulin, the values of both test parameters indicated decreased intestinal permeability at the end of the double blind part of the trial compared with baseline. Differential sugar absorption test decreased in all cases to within the predetermined normal reference range (L/M < 0.035) after colostrum supplementation (Figure 1a). The zonulin level decreased after colostrum supplementation, but remained mostly above the reference limit of >30 ng/mL (5 participants—62.5%).

### 3.1. Double-Blind Phase

#### 3.1.1. Intestinal Permeability (Differential Sugar Absorption Test)

Placebo intake produced no significant difference in the differential sugar absorption test results obtained before and after the supplementation period. Colostrum supplementation produced a significant decrease in intestinal permeability (*p* = 0.01, Figure 1a). The post-intervention to baseline changes in permeability (Δ) within the placebo and the colostrum groups were significantly different (Figure 1b).

#### 3.1.2. Stool Zonulin Concentration

**Figure 2:**
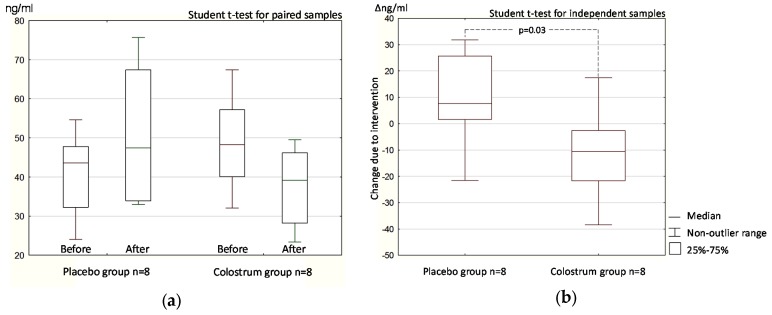
Stool zonulin concentration before and after oral administration of colostrum or placebo (**a**); The change in zonulin concentration after vs. before intervention (Δ) within the colostrum and the placebo groups (**b**). Data from the double-blind placebo-controlled phase of the trial.

Neither placebo nor colostrum supplementation produced a significant difference in zonulin concentration compared with baseline (Figure 2a). While the differences did not reach significance, the zonulin concentration increased in the placebo group and decreased in the colostrum group, which resulted in a statistically significant result (*p* = 0.03) when the post-intervention to baseline changes (Δ) were compared between the placebo and the colostrum groups (Figure 2b).

### 3.2. Open-Label (Crossover) Phase

#### 3.2.1. Intestinal Permeability

**Figure 3:**
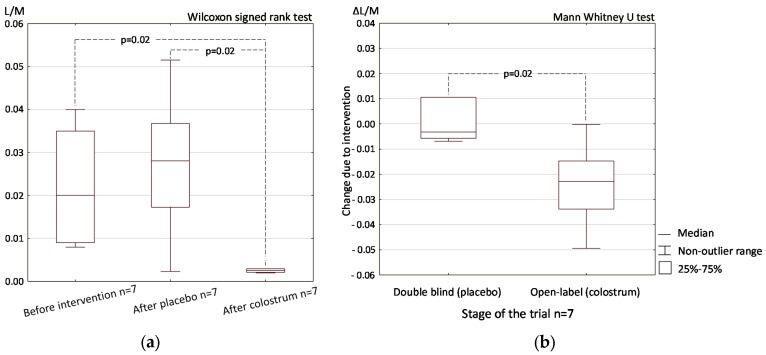
Intestinal permeability measured by differential sugar absorption and expressed as lactulose to mannitol ratio (L/M) in urine on sequential oral administration of placebo and colostrum (**a**); The change in permeability due to supplementation (Δ) with placebo in the double-blind study (day 22 vs. day 0) and with colostrum in the open-label trial (day 44 vs. day 22) (**b**). Data are from open-label (colostrum)/double-blind (placebo) trial phases in the same group of participants.

In the open-label phase, differential sugar absorption on day 44 following colostrum supplementation and on day 22 following placebo supplementation were significantly different (*p* = 0.02, Figure 3a). The results obtained after colostrum supplementation in the open-label phase were also significantly different from the baseline results obtained before the trial began with the intervention on day 0 (*p* = 0.02, Figure 3a).

Comparison of the change (Δ) in differential sugar absorption results following colostrum supplementation in the open-label phase (day 44 vs. day 22) and the change observed in the same group of participants after placebo supplementation in the double-blind phase (day 22 vs. day 0) produced statistically significant results (*p* = 0.02, Figure 3b).

#### 3.2.2. Stool Zonulin Concentration

**Figure 4:**
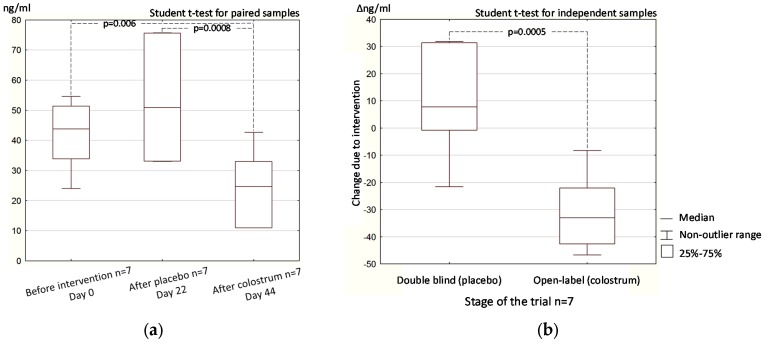
Intestinal (stool) zonulin concentration on sequential oral administration of placebo and colostrum (**a**); The change in zonulin concentration due to supplementation (Δ) with placebo in the double-blind trial (day 22 vs. day 0) and with colostrum in the open-label trial (day 44 vs. day 22) (**b**). Data from open-label (colostrum) and double blind (placebo) trial phases were obtained from the same seven participants.

In the crossover open-label phase of the trial, the change in stool zonulin concentration in the seven participants was parallel to the change in differential sugar absorption. The difference in zonulin concentration after colostrum supplementation in the open-label phase (day 44) and after placebo supplementation in the double-blind phase (day 22) was statistically significant (*p* = 0.0008, Figure 4a). In the open-study, the difference in zonulin concentration after colostrum supplementation (day 44) and before intervention (day 0) was also significant (*p* = 0.006, Figure 4a).

The results of the change in intestinal zonulin concentration (Δ) produced by the intervention with colostrum (open-label phase—day 44 vs. day 22) and the change due to placebo application (double blind phase—day 22 vs. day 0), in the group of the same participants, were significantly different (*p* = 0.0005, Figure 4b).

### 3.3. Colostrum Supplementation Effect in Double-Blind and Open-Label Phases

**Figure 5:**
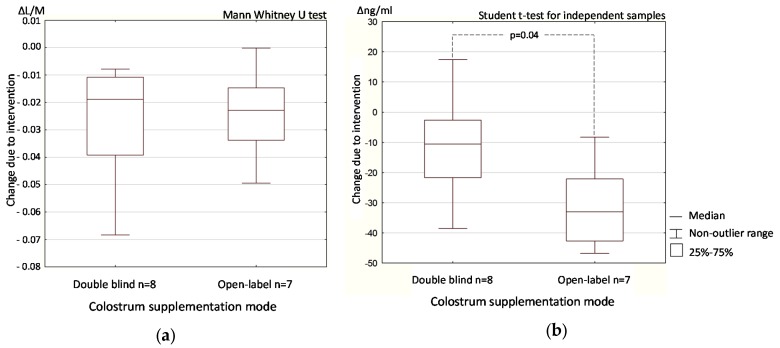
The change due to colostrum supplementation (Δ) in the double-blind vs. open-label phase expressed as the urine lactulose/mannitol ratio (**a**) and stool zonulin concentration (**b**). Data from the open-label (colostrum) and double-blind (colostrum) phase of the trial.

Comparison of double-blind and open-label colostrum supplementation effect as expressed by the within-group difference (Δ) between the post-supplementation vs. baseline test results revealed a statistically significant difference only for zonulin concentration, in which the decrease was greater in the open-label than the double-blind phase (*p* = 0.04, Figure 5b), and not in the L/M differential absorption test (Figure 5a).

## 4. Discussion

To the best of our knowledge, our trial was the first to demonstrate that supplementation with bovine colostrum decreased and mostly restored to normal values two parameters, reflecting intestinal permeability: the lactulose/mannitol ratio in urine and zonulin concentration in stool. Previous human studies demonstrated the beneficial influence of preventive colostrum supplementation on permeability increase due to nonsteroidal anti-inflammatory drug (NSAID) indomethacin treatment or heavy exercise in athletes [28,29,30]. These studies utilized differential sugar absorption test but not zonulin concentration test The rodent studies demonstrated the importance of colostrum as preventive supplement decreasing the direct intestinal mucosa damage resulting from NSAID treatment [22,23].

The L/M test directly reflects, while the stool zonulin concentration test indirectly reflects intestinal permeability. Despite the limited number of study participants [16], the double-blind study produced statistically significant results in both tests (Figure 1 and Figure 2). The results of the open-label part of the study confirmed those obtained in the double-blind part, but may be subject to bias because of the participants’ expectations of a positive effect of supplement administration (Figure 3 and Figure 4).

The results of the differential sugar absorption test, which by its nature more directly reflects the status of intestinal permeability than stool zonulin concentration, were especially interesting. In the double-blind phase of the trial, these results were statistically significant for both the difference before and after colostrum supplementation and for comparison of the change due to intervention (Δ) between the placebo and colostrum groups (Figure 1). All the post-colostrum supplementation results fell within the normal L/M test range even though 75% of the pre-intervention results were above the reference range and some were at double the upper limit of normal (data not shown).

The results of the stool zonulin concentration test in the double-blind phase of the trial were statistically significant only when the changes due to intervention (Δ) within the placebo and colostrum groups were compared (Figure 2). In this phase of the trial, the stool zonulin concentration of all but one participant (12.5%) decreased after receiving colostrum, but was restored to the normal range in only three participants (37.5%).

One of our study goals was designed to determine whether the reported beneficial effect of colostrum on the gut health can be attributed to downregulation of zonulin concentration leading to decreased permeability [20]. It is difficult to draw conclusions on this effect of colostrum on gut permeability based only on our results. Nevertheless, the differential sugar absorption results clearly showed a permeability decrease in response to colostrum supplementation (Figure 1) that was much more evident than that reflected by the change in stool zonulin concentration (Figure 2). Change in sugar absorption seems to be independent to some extent of zonulin downregulation. In four double-blind study participants in the group receiving colostrum (50%), the zonulin level remained above 40 ng/mL, which was above the predetermined upper reference limit of 30 ng/mL. In those participants, the L/M ratio dropped to below 0.01, which was below the predetermined upper reference limit of 0.035. In the double-blind study, the change in L/M ratio after colostrum supplementation reached a higher level of statistical significance than the change in zonulin concentration.

The difference in colostrum post- versus pre-supplementation (Δ) zonulin concentration was significantly larger in the open-label than in the double-blind comparison, whereas no comparable effect was observed for intestinal permeability (Figure 5). This suggests that psychological influence may have influenced zonulin release in the gut. Testing this effect will require further study.

The questionnaire obtained data regarding potential side effects and other health related events were not significantly different between the groups. Moreover, all these events (digestive tract symptoms and upper respiratory tract infections) were found to be reported in similar frequency and variety by the same participants also before the trial has begun. This suggests these events being unrelated to the trial intervention.

There are currently no objective laboratory tests demonstrating colostrum supplementation effect. In this trial, the L/M ratio in urine decreased in all participants with colostrum supplementation. This suggests that a decrease in L/M ratio might be regarded as the first available measure of colostrum supplementation.

In conclusion, our trial demonstrated that colostrum supplementation decreased previously elevated intestinal permeability and was able to restore it to a predetermined normal limit within less than 3 weeks of relatively mild supplementation. Colostrum supplementation also decreased zonulin concentration in the gut, but to a lesser degree than its influence on permeability. It is difficult to draw a conclusion regarding the mechanism leading to decreased permeability. It is possible that in addition to partial influence of decreased zonulin concentration; the normalization of permeability might also result from the powerful healing potential exerted on the intestinal mucosa by colostrum, as was observed in previous studies [22,23]. With regard to the reported prebiotic potential of some of the colostrum components (e.g., bifidogenic effect of lactoferrin) [31], it is also possible that improvement of physiological flora of the gut may have influenced the observed effect on the intestinal permeability in our trial as it was reported in the past by other authors [32].

Our study provides evidence which suggests that plain bovine colostrum, which is a natural and relatively inexpensive supplement, can be responsible for the effective reversal of inappropriately increased intestinal permeability. The use of it might benefit many patients, in whom increased intestinal permeability would ultimately result in various pathologies [7,8,9,10]. Bovine colostrum might also have a preventive role in healthy people and help to restore gut status after use of antibiotics or NSAIDs, both of which can increase intestinal permeability. Among those who may benefit the most from colostrum supplementation are athletes, who not only present tendency to have increased intestinal permeability, but have also been discovered to have increased risk of developing some of the hypersensitivity-based diseases, including allergies [33].

## Figures and Tables

**Table 1:** Anthropometric characteristics of the athletes participating in the trial. No significant differences in these parameters were found between the placebo and supplementation group.

Variable	Mean	SD
Body mass [kg]	89.59	17.85
Waist circumference [cm]	80.32	27.35
Hip circumference [cm]	95.00	30.93
Lean body mass (LBM)	71.79	12.31
Total body water (TBW) [%]	51.71	8.89
Peripheral body fat (PBF) [%]	19.38	5.63
Body mass index (BMI)	27.47	4.10
